# Single injection recombinant vesicular stomatitis virus vaccines protect ferrets against lethal Nipah virus disease

**DOI:** 10.1186/1743-422X-10-353

**Published:** 2013-12-13

**Authors:** Chad E Mire, Krista M Versteeg, Robert W Cross, Krystle N Agans, Karla A Fenton, Michael A Whitt, Thomas W Geisbert

**Affiliations:** 1 Galveston National Laboratory, University of Texas Medical Branch, 301 University Blvd., Galveston, TX, USA; 2 Department of Microbiology and Immunology, University of Texas Medical Branch, 301 University Blvd., Galveston, TX, USA; 3 Department of Microbiology, Immunology, and Biochemistry, University of Tennessee Health Science Center, 858 Madison Ave., Memphis, TN, USA

**Keywords:** Nipah virus, Henipavirus, Vaccine, Vesicular stomatitis virus, Ferret, Fusion protein, Attachment protein, Glycoprotein, Single-injection, Immunity

## Abstract

**Background:**

Nipah virus (NiV) is a highly pathogenic zoonotic agent in the family *Paramyxoviridae* that is maintained in nature by bats. Outbreaks have occurred in Malaysia, Singapore, India, and Bangladesh and have been associated with 40 to 75% case fatality rates. There are currently no vaccines or postexposure treatments licensed for combating human NiV infection.

**Methods and results:**

Four groups of ferrets received a single vaccination with different recombinant vesicular stomatitis virus vectors expressing: Group 1, control with no glycoprotein; Group 2, the NiV fusion protein (F); Group 3, the NiV attachment protein (G); and Group 4, a combination of the NiV F and G proteins. Animals were challenged intranasally with NiV 28 days after vaccination. Control ferrets in Group 1 showed characteristic clinical signs of NiV disease including respiratory distress, neurological disorders, viral load in blood and tissues, and gross lesions and antigen in target tissues; all animals in this group succumbed to infection by day 8. Importantly, all specifically vaccinated ferrets in Groups 2-4 showed no evidence of clinical illness and survived challenged. All animals in these groups developed anti-NiV F and/or G IgG and neutralizing antibody titers. While NiV RNA was detected in blood at day 6 post challenge in animals from Groups 2-4, the levels were orders of magnitude lower than animals from control Group 1.

**Conclusions:**

These data show protective efficacy against NiV in a relevant model of human infection. Further development of this technology has the potential to yield effective single injection vaccines for NiV infection.

## Background

Nipah virus (NiV) and Hendra virus (HeV) represent the highly pathogenic zoonotic agents in the paramyxovirus genus *Henipavirus* with human case fatality rates ranging between 40 and 75% [1]. These viruses are categorized as biosafety level 4 (BSL4) pathogens due to the significant morbidity and mortality associated with disease and the lack of approved vaccines and therapeutics for human use. The primary reservoir for henipaviruses are bats of the genus *Pteropus*[2]; however; the viruses can be transmitted to many mammalian species including humans. Currently, there are two distinct strains of NiV: 1) the Malaysia strain (NiV_M_) discovered in 1999 during an outbreak on pig farms which resulted in spread to humans [3]; and 2) the Bangladesh strain (NiV_B_), which was discovered in India and Bangladesh during 2001 [4]. NiV_B_ has been linked to direct transmission from bats to humans and evidence suggests human to human transmission is possible [5].

The near annual outbreaks of NiV_B_ with high case fatality rates [6] underscores the urgent need for effective vaccines and therapeutics. To date, there have been four experimental preventive candidate vaccines against henipaviruses evaluated in animal models. Vaccinia and canarypox viruses encoding the NiV_M_ glycoproteins have shown protection against NiV_M_ in hamsters and pigs [7,8]. A recombinant adeno-associated vaccine expressing the NiV_M_ G protein completely protected hamsters against homologous NiV_M_ challenge and protected 50% of animals against heterologous HeV infection [9]. In addition, a recombinant subunit vaccine based on the HeV G protein (sG_HeV_) completely protects small animals against lethal HeV and NiV_M_ infection [10-13] and more recently was shown to be efficacious in the robust African green monkey model of NiV_M_ infection [14]. Though very promising, the sG_HeV_ vaccine requires a prime-boost strategy to confer protection whereas a single-injection vaccine would be particularly beneficial during outbreaks where there is little time to employ lengthy vaccination regimens.

Single-injection recombinant vesicular stomatitis virus (rVSV) vectors have been developed as vaccine candidates against many important human pathogens such as papillomavirus [15,16], human immunodeficiency virus (HIV) [17-19], influenza virus [20], measles virus [21,22], respiratory syncytial virus [23,24], severe acute respiratory syndrome coronavirus [25], chikungunya virus [26], and hemorrhagic fever viruses such as Lassa, Ebola, and Marburg [27]. Single-cycle replication rVSVs have been developed against NiV and have shown strong immunogenicity in mice vaccinated with rVSVs expressing either the NiV_M_ fusion protein (F) or the NiV_M_ attachment protein (G) as high neutralizing antibody titers were generated [28]. These vaccine vectors were just recently shown to provide homologous protection in the hamster model of NiV_M_ infection [29].

Here, we developed alternative rVSV vaccine vectors expressing either the NiV_B_ F or NiV_B_ G proteins. These vaccines were evaluated 28 days after a single dose vaccination in the NiV_M_ ferret model, which along with the African green monkey, most faithfully recapitulates human disease [30-32]. Each group of specifically vaccinated ferrets were protected from NiV_M_-induced disease while the non-specifically vaccinated ferrets succumbed to NiV_M_ infection. To date, this is the first study to protect ferrets from NiV infection using a single-injection vaccine.

## Results

### Recovery of rVSVΔG-NiV_B_/glycoprotein vectors

**Figure 1:**
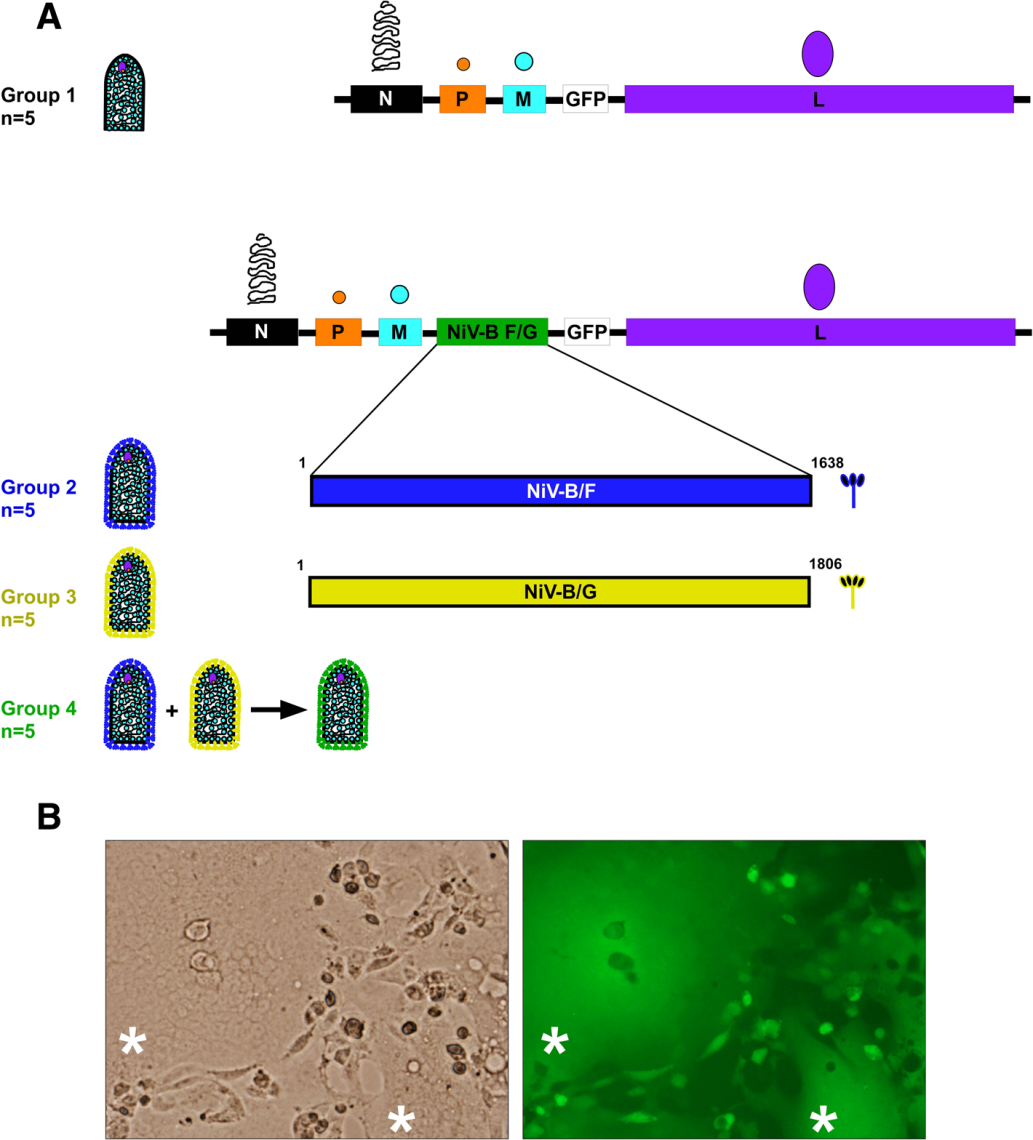
**Schematic rVSV NiV**_**B **_**vaccine genomes and groups. (A)** Group 1: rVSV-ΔG-GFP expressing no glycoprotein; produces ‘bald’ virions, Group 2 G_Ind_*rVSV-NiV_B_ /F-GFP expressing the NiV_B_ F protein (blue); produces virions with F on the surface, Group 3 G_Ind_*rVSV-NiV_B_ /G-GFP expressing the NiV_B_ G protein (yellow); produces virions with G on the surface, Group 4 made from co-infection in Vero cells resulting in a combination of rVSV-NiV_B_ /F-GFP and rVSV-NiV_B_ /G-GFP virions expressing the NiV_B_ F and G proteins in the same cells; produces single-cycle infectious virions with NiV_B_ F and G proteins on the cell surface (green). **(B)** Phase and fluorescence microscopy of Vero cells infected with virions from Group 4 displaying multinucleated syncytia cells * and cells positive for GFP expression.

To investigate the protective efficacy of rVSV NiV_B_ vaccine vectors against heterologous NiV_M_ challenge in ferrets, we first developed and recovered two rVSVΔG constructs expressing the NiV_B_ F protein rVSV-ΔG-NiV_B_/F-GFP (Figure 1A, blue) or NiV_B_ G protein rVSV-ΔG-NiV_B_/G-GFP (Figure 1A, yellow) using reverse genetics. Propagation of these vectors requires VSV glycoprotein (G_Ind_) complementation (G_Ind_*) of viruses where G_Ind_ is provided in trans during infection [33]. G_Ind_* complementation allows for single-cycle replication of vectors and results in expression of the NiV_B_ glycoproteins and the production non-infectious virions containing either glycoprotein. As seen previously with similar NiV_M_ rVSV vectors [28], co-infection with G_Ind_* rVSV-ΔG-NiV_B_/F-GFP and G_Ind_* rVSV-ΔG-NiV_B_/G-GFP produced infectious virions (rVSV-ΔG-NiV_B_/F/G-GFP) containing either genome with the NiV_B_ F and NiV_B_ G proteins incorporated in the virion envelope (Figure 1A, Group 4 green-spiked virion) as evidenced by the ability of these virus preparations to infect Vero cells (Figure 1B, *) and the inability of the rVSV-ΔG-NiV_B_/F-GFP and rVSV-ΔG-NiV_B_/G-GFP to infect cells without G_Ind_* complementation (negative data not shown). The rVSV-ΔG-NiV_B_/F/G-GFP virus stocks were able to reach titers of up to 3 × 10^8^ PFU/ml and infection of Vero cells with these stocks resulted in syncytia formation (Figure 1B). While this vaccine preparation could undergo a single round of replication, it could not be passaged further without G_Ind_* complementation as was observed with the rVSV-ΔG-NiV_B_/F-GFP and rVSV-ΔG-NiV_B_/G-GFP vaccine preparations.

### Immunization of ferrets and examination of the humoral immune response

**Figure 2:**
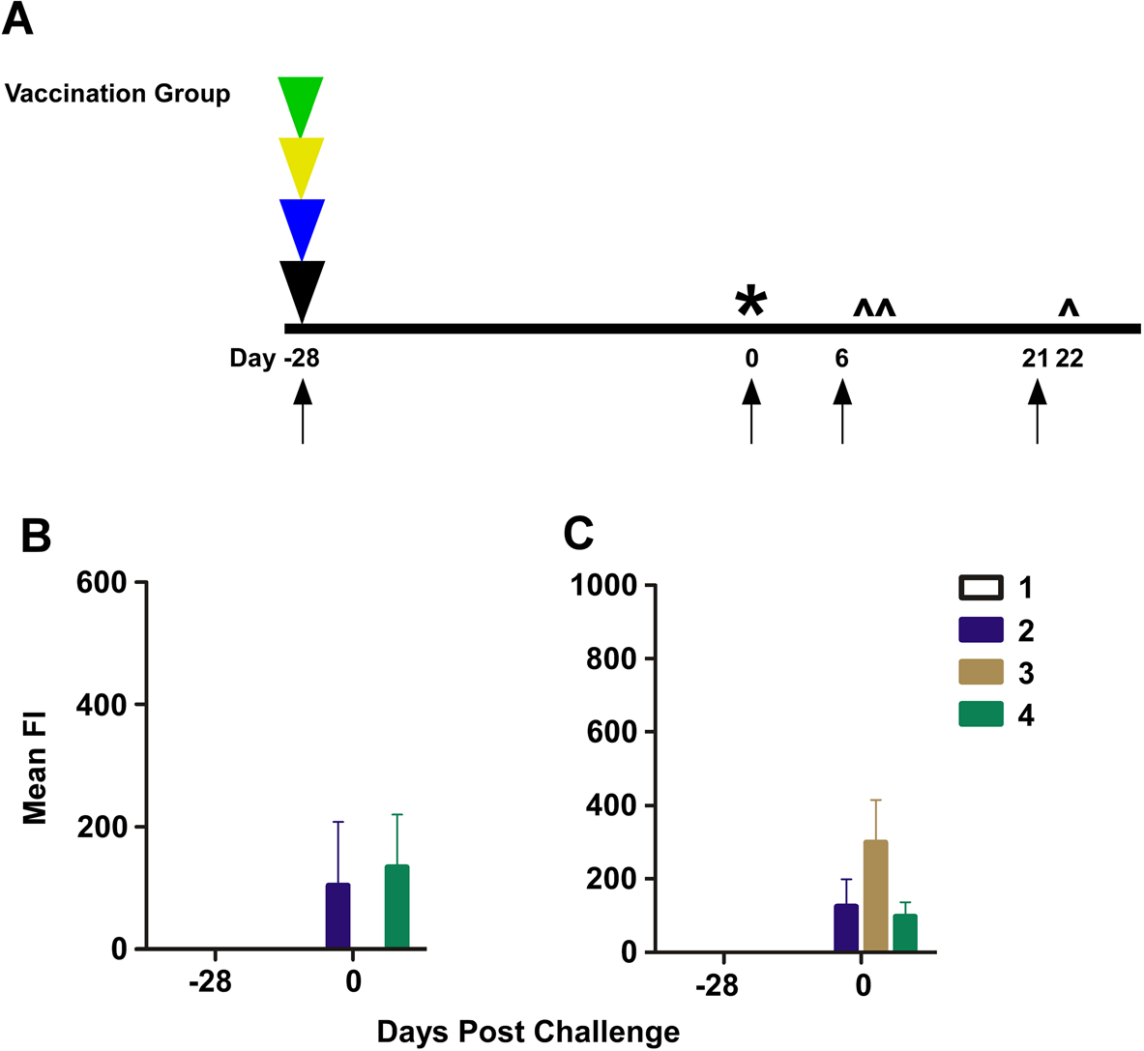
**NiV vaccine study design and circulating IgG post vaccination. (A)** Flow chart showing the days of vaccination (triangles), days of sampling (arrows), day of challenge (*),terminal succumbing to infection (^, day 7 or 8), and day of necropsy (^, day 22). Black triangle, Group 1; blue triangle Group 2; yellow triangle Group 3; green triangle Group 4 vaccination. **(B and C)** Detection of specific anti-NiV F **(B)** and anti-NiV G **(C)** IgG antibodies circulating in vaccinated ferrets. Mean fluorescence intensities (Mean FI) are shown on the y-axis and represent binding of specific IgG_._ Error bars represent the s.d. of fluorescence intensity across 100 beads for each sample.

Four groups of ferrets (Figure 1A) received a single vaccination of rVSV vectors as follows: Group 1 served as the nonspecific vaccine control group and received G_Ind_* rVSV-ΔG-GFP; Group 2 received G_Ind_* rVSV-NiV_B_/F-GFP; Group 3 received G_Ind_* rVSV-NiV_B_ /G-GFP; and Group 4 received the rVSV-ΔG-NiV_B_/F/G-GFP (Figure 2A, triangles). Serum collected from each animal on the day of vaccination (day -28) and just before challenge (day 0) was analyzed for circulating IgG to the NiV F and NiV G proteins by microsphere assay [30]. As expected, we did not detect NiV F-specific Ig in sera before vaccination (Figure 2B, day -28). We were able to detect Ig directed at NiV F in sera of the Group 2 and 4 vaccinated cohorts 28 days after vaccination but not in the ferrets from Groups 1 and 3 (Figure 2B, day 0). Similar to the analysis of NiV F-specific circulating IgG in vaccinated ferrets, we were unable to detect IgG directed to NiV G before vaccination (Figure 2C, day -28) and were able to detect anti-NiV G IgG 28 days post vaccination (Figure 2C, day -28). Animals in Group 3 had higher levels of circulating anti-NiV G IgG when compared to Group 4 and surprisingly we were able to detect anti-NiV G IgG in Group 2 animals although not to the levels of the NiV_B_ G-specifically vaccinated Group 3 animals (Figure 2C, day -0). These results suggested that the animals in Groups 2-4 and not Group 1 had generated a humoral immune response to the rVSV-ΔG-NiV_B_ antigens delivered in a single injection.

### NiV challenge of vaccinated ferrets

**Figure 3:**
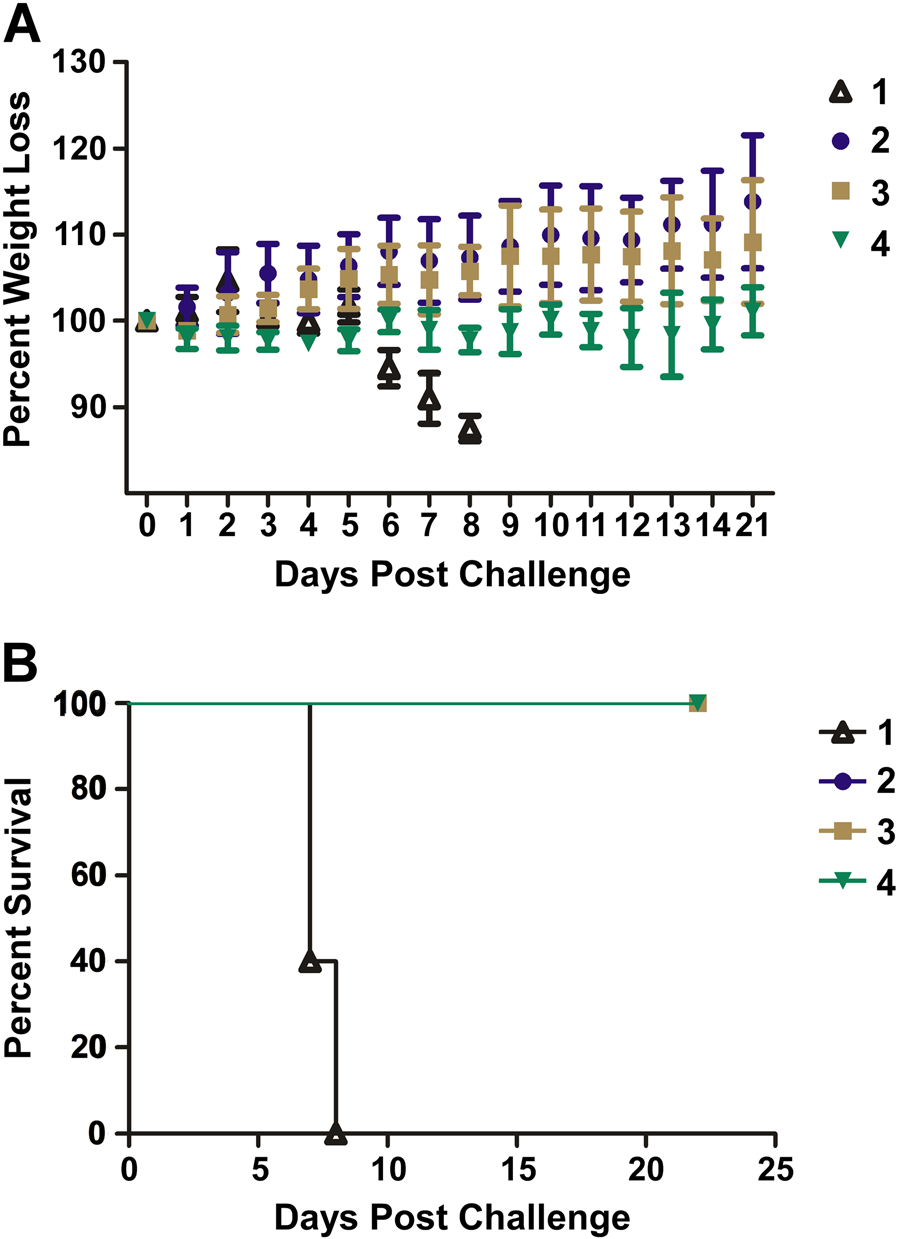
**Protection from NiV**_**M **_**mediated disease. (A)** Weight curves of vaccinated ferrets are shown and represent the average percent weight of animals post NiV_M_ challenge in comparison to day 0. Error bars represent the s.d. **(B)** Kapplan-Meier survival curve for each vaccine group post NiV_M_ challenge.

**Figure 4:**
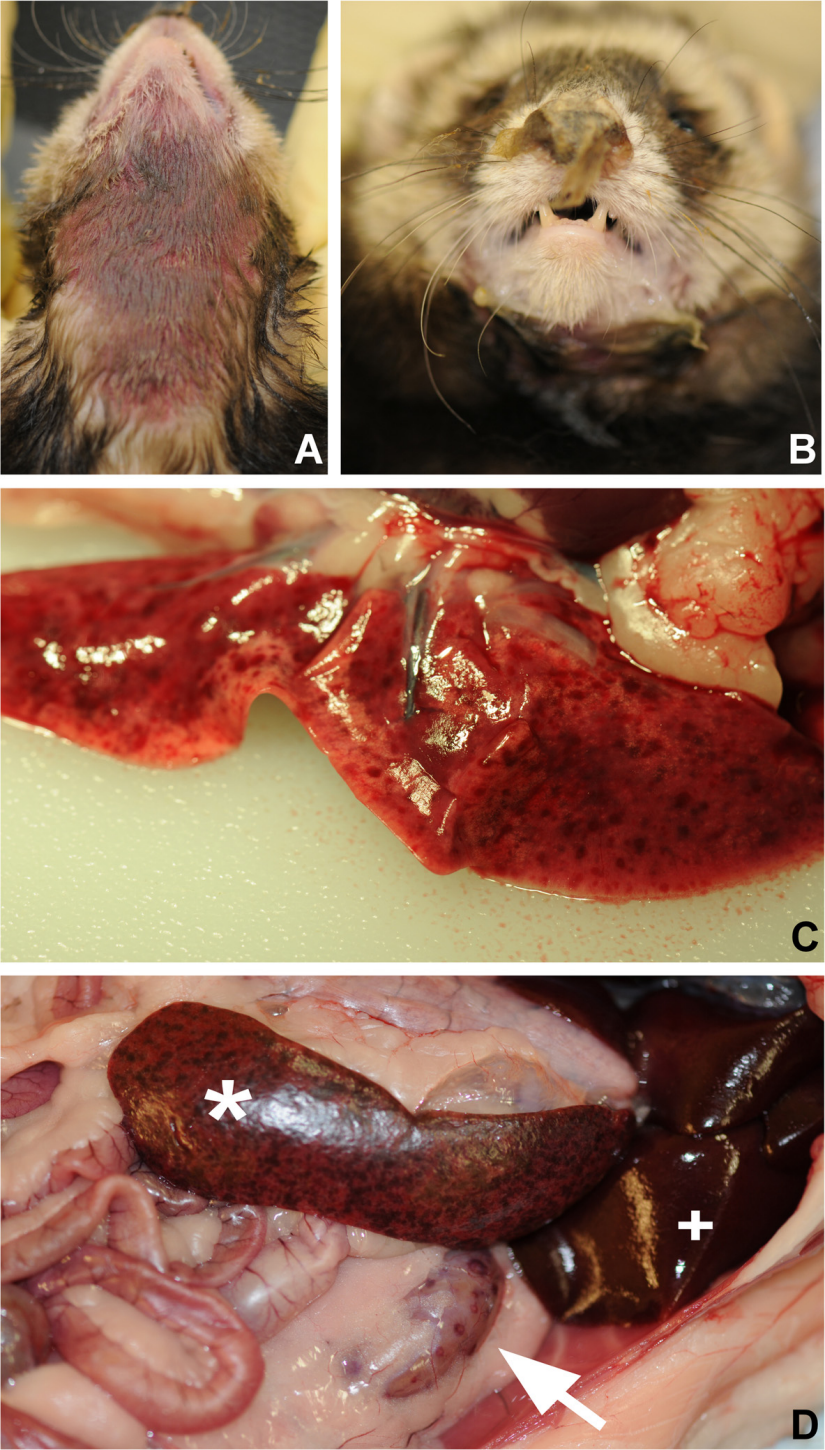
**Gross pathology of ferrets succumbing to NiV**_**M **_**mediated disease.** Representative gross pathology from animals in Group 1 challenged with NiV_M_. **(A)** Ventral cervical subcutaneous hemorrhage with edema. **(B)** Crusting serous nasal discharge. **(C)** Diffuse hemorrhagic interstitial pneumonia. **(D)** Splenomegaly with multifocal necrosis (*), diffuse reticulation of the liver (+), and multifocal renal hemorrhage (white arrow).

To determine whether vaccination with the rVSV-ΔG-NiV_B_ vectors could prevent NiV_M_ disease course in ferrets, we challenged ferrets intranasally with a lethal challenge dose of NiV_M_ on day 0 (Figure 2A, *). The animals were closely monitored over the course of 22 days post challenge (p.c.) for clinical signs of illness. The specifically vaccinated animals in Groups 2-4 did not lose weight over the course of the study (Figure 3A) and were 100% protected against NiV_M_ (Figure 3B), while the animals in the non-specifically vaccinated Group 1 succumbed to infection on days 7 or 8 (Figure 2A, **^**), respectively (Figure 3B, Table 1). Clinical scores were recorded each day after challenge for each animal using a scoring system based on coat grooming, social behavior, and provoked behavior. The clinical scores for each animal correlated with the survival data as seen with the mean clinical score for each animal in the vaccinated groups having no score on any day p.c. versus the animals in Group 1 having clinical scores on days 5 to 8 p.c. Clinical signs in response to NiV_B_ infection were more dramatic for the animals in Groups 1 when compared to the animals in the other three groups (Table 1). In all animals that succumbed to NiV_M_ infection, the gross pathologic findings included varying severity of dehydration, ventral cervical subcutaneous hemorrhage with edema (Figure 4A) and crusting serous nasal discharge (Figure 4B). The internal gross pathologic findings from animals in Group 1 included varying severity of heavy, wet, diffusely mottled with dark pin point foci throughout the pulmonary parenchyma, multifocal pin point raised foci throughout the renal parenchyma, mottling of the spleen with diffuse splenomegaly, and diffuse reticulation of the liver. Each animal had diffuse hemorrhagic interstitial pneumonia (Figure 4C) and splenomegaly with multifocal necrosis (pic 4D, *). Additionally, diffuse reticulation of the liver (Figure 4D, +) and multifocal renal hemorrhage (Figure 4D, arrow) were noted. There were no external or internal gross pathologic findings of note in any of the Group 2-4 animals at the study endpoint (Figure 2A, day 22 **^**).

**Table 1:** Clinical description and outcome of Nipah virus challenged ferrets

**Group-subject no.**	**Clinical illness**^**a**^	**Viral**^**b **^**load**	**Clinical and gross pathology**
1-1	Fever (d5-7); Depression (d6-7); lethargy (d6-7); loss of appetite (d6-7); labored breathing (d6-7). Animal euthanized on d7.	**++/6, ++/7**	Thrombocytopenia (d7); Lymphopenia (d6-7); >3-fold increase in BUN (d7); rash on ventral surface of neck; lungs with severe congestion and hemorrhage of all lobes; enlarged spleen; darkened liver, edema of left kidney capsule.
1-2	Fever (d5); depression (d6-8); lethargy (d6-8) loss of appetite (d6-8); labored breathing (d6-8); dehydration (d8), ocular and nasal discharge (d7-8); Hind limb paresis (d8) Animal euthanized on d8.	**++/6, ++/8**	Thrombocytopenia (d8); Lymphopenia (d6,8); >3-fold increase in BUN (d8); hypoalbuminemia (d6,8); ecchymotic rash on ventral surface of neck; lungs with severe congestion and hemorrhage of all lobes; enlarged spleen; darkened liver.
1-3	Fever (d4-7); depression (d5-7); lethargy (d5-7); loss of appetite (d5-7); labored breathing (d6-7); ocular and nasal discharge (d6-7); Hind limb paresis (d7). Animal euthanized d7.	**++/6, ++/7**	Thrombocytopenia (d6-7); Lymphopenia (d6-7); hypoalbuminemia (d6-7); ecchymotic rash on ventral surface of neck; lungs with severe congestion and hemorrhage of all lobes; enlarged spleen; darkened liver.
1-4	Fever (d5-7); Depression (d6-8); lethargy (d7-8); loss of appetite (d6-8); labored breathing (d6-8); hind limb tremors (d8). Animal euthanized on d8.	**++/6, ++/8**	Thrombocytopenia (d8); Lymphopenia (d6,8); hypoalbuminemia (d8); lungs with severe congestion and hemorrhage of all lobes; enlarged spleen; darkened liver.
1-5	Fever (d5-7); Depression (d6-7); lethargy (d7); loss of appetite (d5-7); dehydration (d7); labored breathing (d6-7). Animal euthanized on d7.	**++/6, ++/7**	Thrombocytopenia (d7); Lymphopenia (d6-7); hypoalbuminemia (d7); lungs with severe congestion and hemorrhage of all lobes; enlarged spleen; darkened liver.
2-1	None		None
2-2	None	**+/6**	None
2-3	None		None
2-4	None		None
2-5	Mild fever (d7)	**+/6**	None
3-1	None		None
3-2	None		None
3-3	None	**+/6**	None
3-4	None		None
3-5	None	**+/6**	None
4-1	None		None
4-2	None		None
4-3	None	**+/6**	None
4-4	None	**+/6**	None
4-5	None		None

^a^Animals euthanized due to complications from NiV-induced disease.

^b^qRT-PCR positive blood/day p.c.: +, ≤ 5 log_10_ NiV_M_ GEq/ml of blood; ++, ≥ 5 log_10_ NiV_M_ GEq /ml of blood.

### Histopathological and immunohistochemical analysis of NiV_M_-infected ferrets

**Figure 5:**
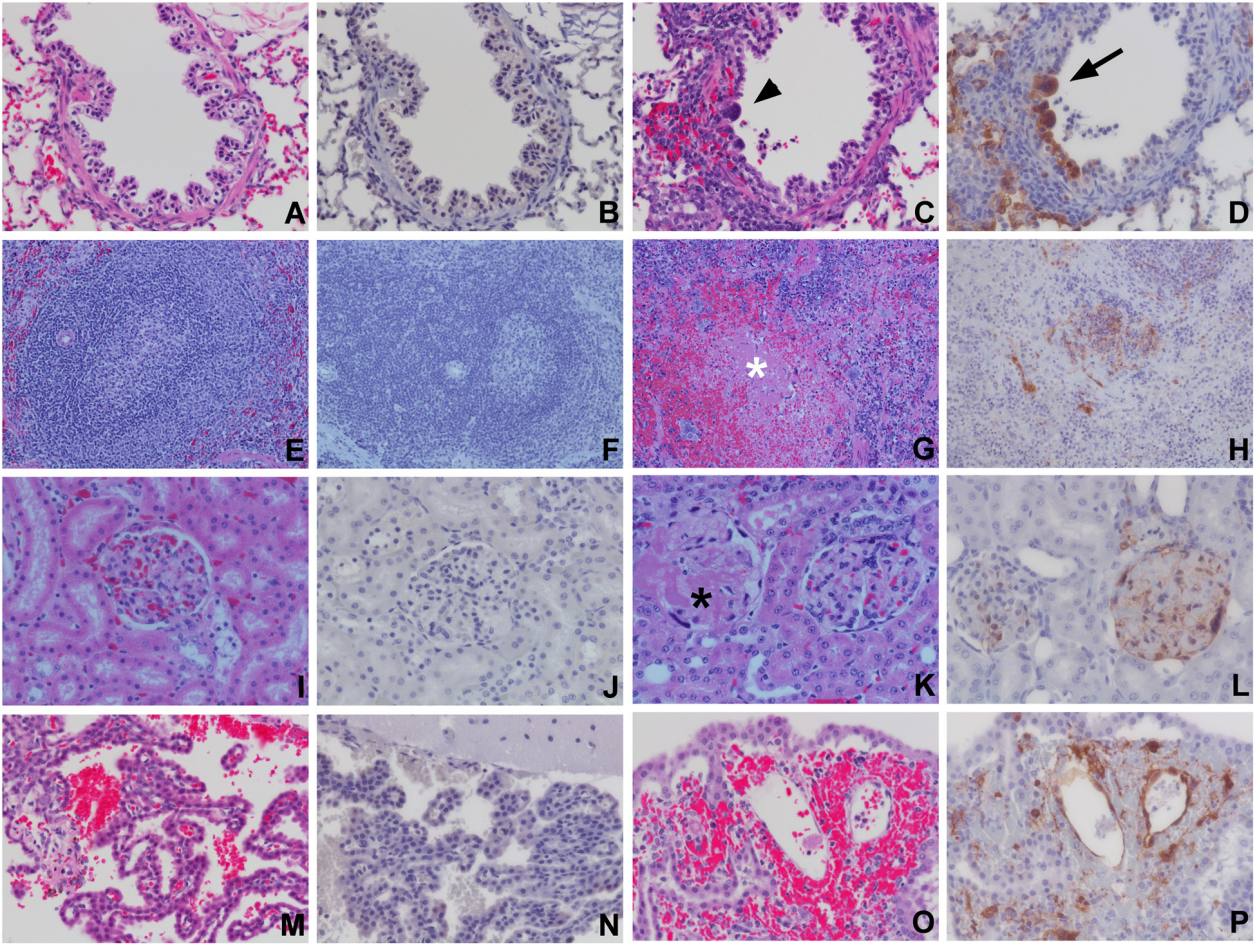
**H&E and immunohistochemistry of ferret tissues.** Representative H&E stained tissues **(A, C, E, G, I, K, M, and O)** and immuno-histochemistry of tissues labeled with a NiV N protein-specific polyclonal rabbit antibody **(B, D, F, H, J, L, N, and P)**. Tissues from representative vaccinated ferrets from groups 2-4; lung **(A, B)**, spleen **(E, F)**, kidney **(I, J)**, and brain, choroid plexus **(M, N)**. Tissues from representative control Group 1 ferrets; lung **(C, D)**, spleen **(G, H)**, kidney **(K, L)**, and brain, choroid plexus **(O, P)**. **C**, (arrow head) Respiratory epilthelial syncytia; **D**, (arrow) NiV antigen-positive respiratory epithelial syncytia, **G**, (white asterisk) lymphoid depletion in spleen; **K**, (*) fibrin deposition in glomerular tuft of kidney. Images taken: lung 40x, spleen 20x, kidney 40x, brain 40x.

Tissues examined from animals in Groups 2-4 had no significant histologic findings (Figure 5A,E,I, and M) and were devoid of NiV antigen (Figure 5B,F,J, and N). In contrast, tissues examined from ferrets in control Group 1 had substantial histologic findings which included mild to moderate interstitial pneumonia with marked congestion, endothelial syncytial cell formation, and respiratory epithelial syncytial cell formation (Figure 5C, arrow head). Severe lymphoid depletion, necrosis, hemorrhage, fibrin deposition and syncytial cell formation were observed throughout the spleen (Figure 5G). Glomerular tufts in the kidney had multifocal segmental to global fibrin deposition and endothelial syncytial cell formation (Figure 5K). Ferrets in control Group 1 also had detectable NiV antigen systemically, with the endothelium, scattered mononuclear cells, and syncytial cells (endothelial and epithelial) of the lung (Figure 5D), spleen (Figure 5H), kidney (Figure 5L), and brain (Figure 5P) having strong immunolabeling for NiV antigen.

### NiV load

**Figure 6:**
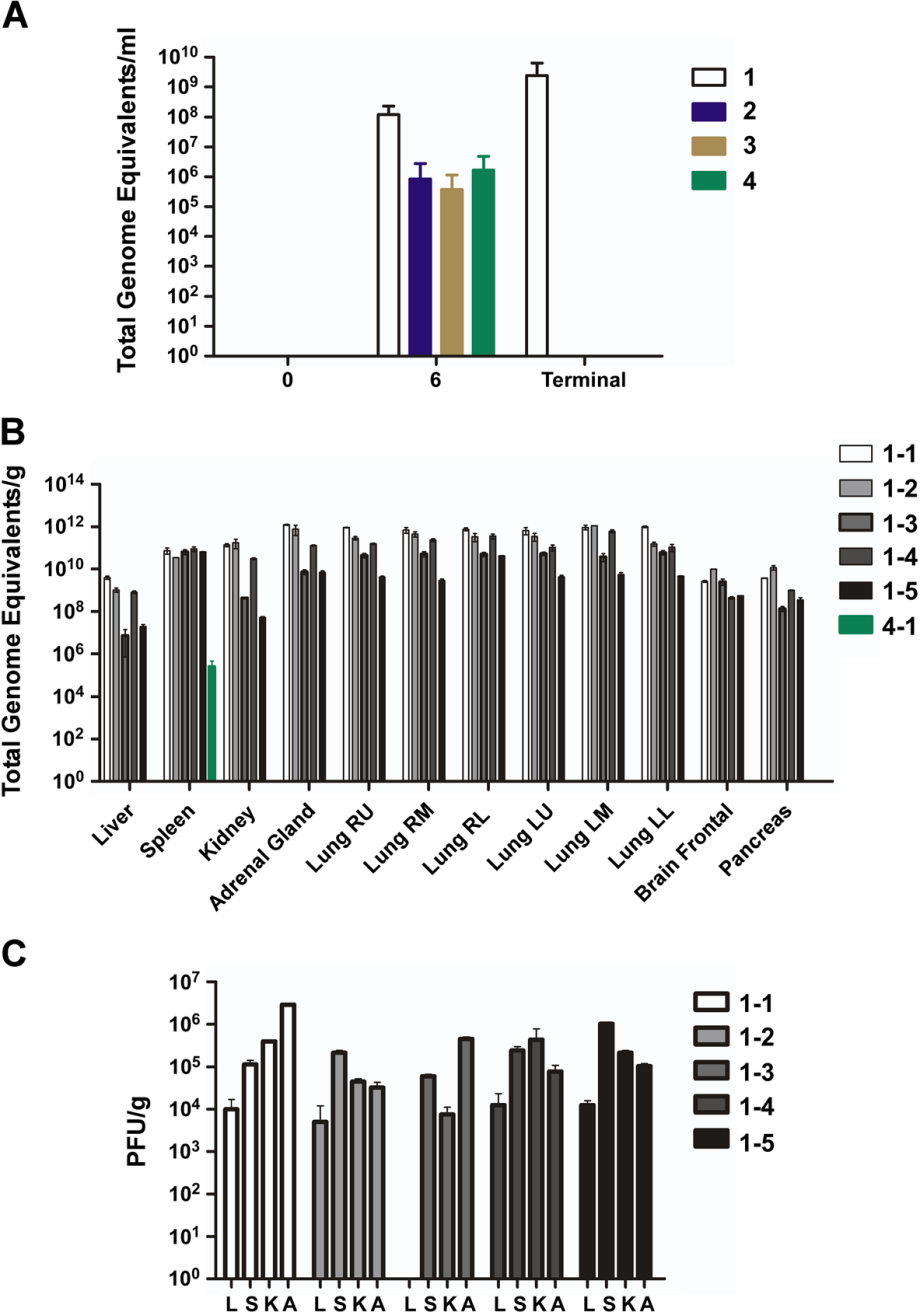
**Viral load in NiV**_**M **_**challenged ferrets.** Viral load in ferrets as detected by genome equivalents (GEq) by qRT-PCR from **(A)** blood as GEq/ml and **(B)** from tissues a GEq/g (Animal 4-1 was negative for NiV_M_ antigen in spleen). Right upper (R.U.), right middle (R.M.), right lower (R.L.), left upper (L.U.), left middle (L.M.), left lower (L.L.), lymph node (LN). **(C)** PFU/g of NiV_M_ isolated from tissues of ferrets in Group 1. Liver (L), spleen (S), kidney (K), adrenal gland (A). Error bars represent the s.d.

To determine if there was NiV_M_ replication in animals p.c., viremia was assessed by virus isolation from serum (negative data not shown) and by qRT-PCR on whole blood samples (Figure 6A). NiV_M_ genome equivalents (GEq) were detected in all blood samples from day 6 p.c. While we detected NiV_M_ GEq for all animals on day 6, the Group 1 animals had over 100 fold more detectable GEq and an increase in GEq from terminal bleeds on day 7 or 8 (Figure 6A). However, none of the specifically vaccinated animals had any detectable NiV_M_ RNA in terminal blood samples on day 21 (Figure 6A). NiV_M_ RNA was also detected systemically in the tissues of all animals that succumbed to infection in control Group 1 and in the spleen of one animal from Group 4 on the study end date (Figure 6B), though no NiV_M_ antigen was detected by immunohistochemistry (data not shown), whereas NiV_M_ RNA was not detected in the tissues of the remaining animals in Groups 2-4. Additionally, we were able to isolate NiV_M_ from all tissues sampled in all animals from control Group 1, with the exception of the liver for animal 1-3 (Figure 6C). Overall, the level of detection of NiV_M_ RNA in tissues and blood correlated with outcome gross pathology, and histology (Figure 4, Figure 5, and Table 1) for each animal.

### Neutralizing NiV_M_ antibody titers pre and post challenge with NiV_M_

**Figure 7:**
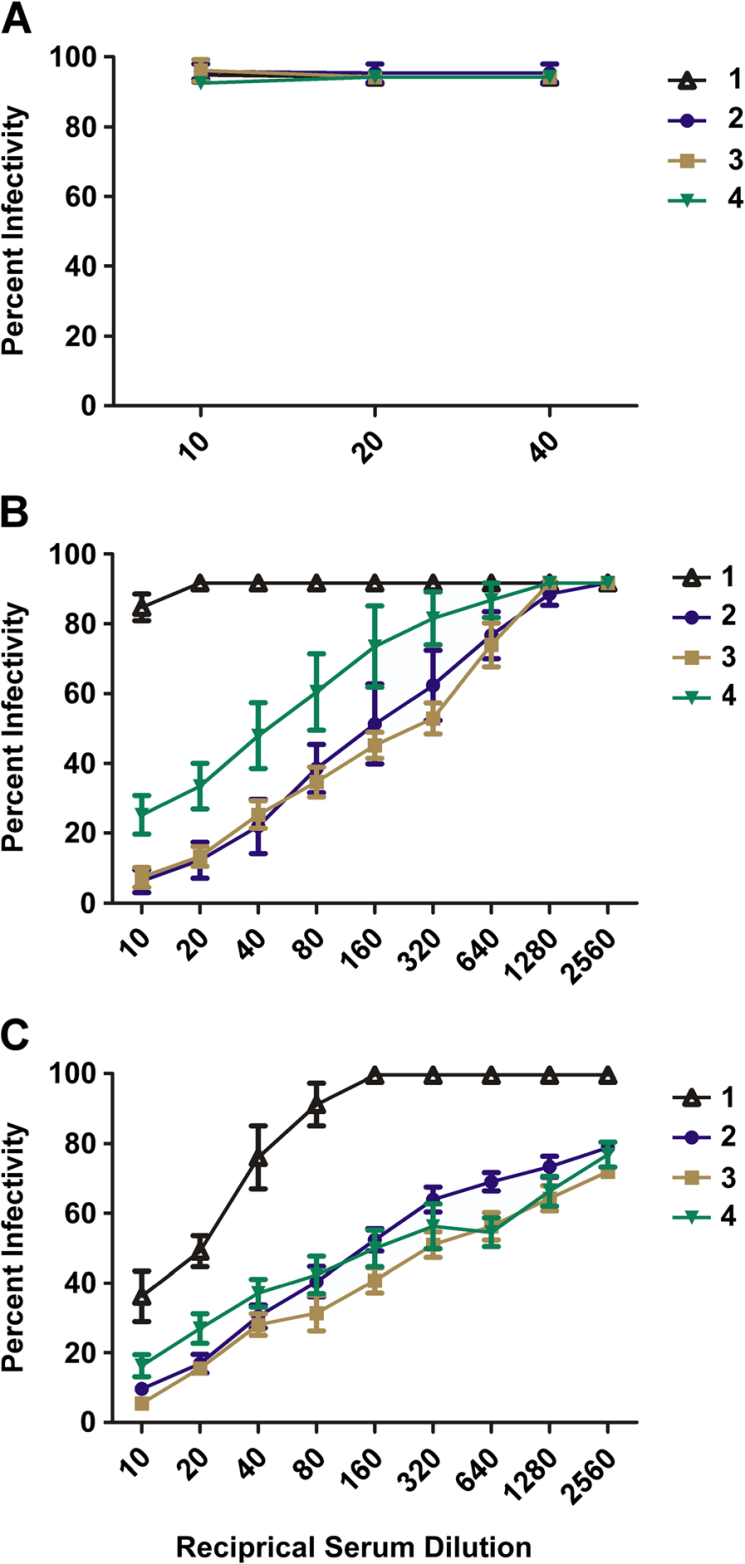
**Reciprocal NiV**_**M **_**serum neutralizing antibody titers.** Neutralizing antibody titers which reduce virus infectivity are shown for all groups on the **(A)** day of vaccination showing a lack of neutralizing antibody for all groups, **(B)** on the day of challenge showing neutralizing antibodies for all groups except Group 1, and **(C)** on the terminal bleed days showing high levels of neutralizing antibodies for Groups 2-4 and low neutralizing antibodies for Group 1. Error bars represent the s.d.

To further address the antibody response to rVSV-ΔG-NiV_B_ vaccination and after NiV_M_ challenge, we characterized the circulating antibodies before and after vaccination for their neutralizing activity using a plaque reduction neutralization titer (PRNT_50_) assay. All four groups lacked neutralizing antibody titers before vaccination (Figure 7A). On the day of challenge, animals from control Group 1 did not have neutralizing antibody titers against NiV_M_ whereas all ferrets from Groups 2-4 had neutralizing antibodies against NiV_M_ (Figure 7B), although it appeared that Groups 2 and 3 had higher titers than Group 4. Neutralizing antibody titers were also assessed for all animals from terminal bleeds on the day of death (day 7 or 8) or at day 21. All animals that succumbed to NiV_M_ challenge in Group 1 had consistently lower neutralizing antibody titers against NiV_M_ when compared to the neutralizing antibodies found in animals from Groups 2-4 (Figure 7C).

## Discussion

While significant progress on a veterinary vaccine for one henipavirus, HeV, has been made [34], the development of effective human vaccines and antiviral drugs for high consequence pathogens such as NiV is a much slower and complicated process. In particular, the restriction of infectious NiV work to BSL-4 containment has hampered progress. Conventional clinical trials with viruses such as NiV are not practical. To address the development of countermeasures for exotic pathogens such as NiV the FDA implemented the Animal Efficacy Rule in 2002. This rule specifically applies to the development of countermeasures when human efficacy studies are not possible or ethical. Briefly, this rule permits the evaluation of vaccines or therapeutics using data generated from studies performed in animal models that faithfully recapitulate human disease. There are currently at least eight animal models for NiV including the IFNAR-KO mouse model [35]; the guinea pig model [36]; the pig model [8,37-39]; the cat model [10,11]; the golden Syrian hamster model [7,40-42]; the ferret model [30,43]; and two nonhuman primate models, one in squirrel monkeys [44] and the other in African green monkeys [32]. While each of these models shares at least one or more aspects seen in human pathogenesis and can contribute to vaccine and therapeutic testing, the two animal models which completely emulate the pathogenesis seen in human cases to date are the ferret model and the African green monkey model. These models recapitulate what is seen during human infection with severe respiratory and neurologic disease and a generalized vasculitis.

Based on this knowledge, the ferret model was chosen for the initial evaluation of our rVSV-ΔG-NiV_B_-F/G-GFP vectors. This model should be considered as the initial small animal screening model for vaccines against NiV, especially since the recombinant sG_HeV_ protein vaccine has been shown to be efficacious in ferrets [12] and African green monkeys [14]. While sG_HeV_ vaccine has proven to have utility against NiV infection in these two animal models, the vaccine regimen requires two doses using a prime-boost strategy with the two adjuvants CpG oligodeoxynucleotide (ODN) 2006 and Alhydrogel included in the vaccine formulation [14]. Here, we evaluated rVSV-ΔG-NiV_B_-F/G-GFP vectors as single-injection vaccines against lethal NiV_M_ challenge in ferrets.

Non-specifically vaccinated control animals in Group 1 did not produce anti-NiV F or anti-NiV G IgGs, did not produce significant neutralizing antibody titers against NiV_M_, and thus were not protected from NiV_M_-induced disease as they displayed clinical symptoms and pathology consistent with NiV_M_ infection, and had viral RNA and infectious virus isolated systemically. Animals in Group 2 vaccinated with G_Ind_* rVSV-ΔG-NiV_B_/F-GFP generated anti-NiV F IgGs (and possible cross-reactive NiV G IgG, Figure 2C) and neutralizing antibody titers against NiV_M_ and were protected from NiV_M_-induced disease with one animal (Table 1, 2-5) having a low-grade fever on day 7 p.c. Similarly, ferrets in Group 3 vaccinated with G_Ind_* rVSV-ΔG-NiV_B_/G-GFP generated anti-NiV G IgGs and neutralizing antibody titers against NiV_M_ and were protected from NiV_M_-induced disease. Using vectors that were independent of G_Ind_* complementation, ferrets in Group 4 vaccinated with rVSV-ΔG-NiV_B_-F/G-GFP generated anti-NiV F and anti-NiV G IgGs and neutralizing antibody titers against NiV_M_ and were also protected from NiV_M_-induced disease. The observation that the percent weight growth was less than Group 2 and 3 (Figure 3A) could have been due to the social housing of these animals where it is possible that certain members of the cohort were out competed for extra food as some animals in this group reached percent weight growth of animals from the other groups. While the protection afforded to Groups 2-4 did not appear to be sterile as virus replication could be detected by the presence of viral RNA in whole blood at day 6 p.c., it was only detected in two animals per group versus all animals in Group 1 (Table 1) and the mean virus replication was 100 times lower than what was detected in animals from Group 1 (Figure 6A).

The observation of anti-NiV F antibodies circulating in the rVSV-ΔG-NiV_B_/G-GFP vaccinated ferrets was interesting considering our experience with the Bioplex assay and sG_HeV_ vaccinated ferrets and African green monkeys where we have never detected cross-reactivity against NiV_M_ F from G vaccinated animals. One possible explanation is that the antibodies produced in response to the NiV_B_ G results in the formation of some antibodies that can recognize NiV_M_ F since the target antigen in the Bioplex assay is NiV_M_ F. Our experience with the sG_HeV_ vaccine may not be applicable here as this cross-reactivity may be a single-cycle replicating vaccine observation. While not within the scope of this study, the further examination of the avidity of these antibodies toward NiV_M_ F is warranted as this rVSV-NiV vaccine platform is developed.

As it is difficult to determine which vaccine platform would be best for the human population against deadly pathogens such as NiV, it is important to develop and test a number of platforms to combat a potential outbreak. A preventive vaccine would be important for several populations: 1) the general population during NiV outbreaks particularly in endemic areas in India and Bangladesh; 2) healthcare workers and family members involved in patient care and management in endemic regions; 3) personnel involved in outbreak response missions; 4) laboratory workers conducting research on NiV; and 5) military and other service personnel susceptible to the use of NiV as a biological weapon. While multi-dose vaccine regimens would be feasible for laboratory and healthcare workers and some military personnel in stable settings with defined risk, an outbreak setting or a case of deliberate release would require rapidly conferred protection with a single administration. Here we describe the protection of ferrets from NiV_M_-induced disease using single-injection rVSV vaccine vectors expressing the NiV_B_ F and/or NiV_B_ G proteins. To date, this is the first study to show efficacy of a vaccine against NiV using a single administration in the ferret model. Based on the success of this study, these vectors should be evaluated further in the African green monkey model against NiV_B_ challenge as this strain appears to be the most pathogenic in humans [6].

## Methods

### Plasmid design and construction

RNA was isolated from NiV_B_ virus stocks (NiV_B_ #200401066 was obtained from a patient from the 2004 outbreak in Bangladesh (kindly provided by Dr. Thomas Ksiazek) using approved protocols at biosafety level 4 (BSL-4) in the Galveston National Laboratory (GNL) at the University of Texas Medical Branch (UTMB). The RNA was used to make cDNA with gene specific primers and the genes were amplified using gene specific primers (NiV_B_ F or G) containing MluI and NheI restriction sites at the 5′ and 3′ ends respectively. The PCR amplified products and pΔG-VSV-2.6 plasmid were restriction digested with MluI and NheI and gel purified. Purified products and vector were ligated and positive colonies were screened and sequenced for positive constructs.

### rVSV vaccine vectors and challenge virus

The rVSV NiV_B_ vaccines (rVSV-ΔG-NiV_B_/F-GFP and rVSV-ΔG-NiV_B_/G-GFP) and rVSV-ΔG-GFP were recovered using methods previously described [33]. The rVSVΔG viruses were propagated on BHK-21 cells transfected with 2 μg of pCAGGS-G_Ind_ expressing the VSV glycoprotein (G_Ind_) and titered as previously described [33]. Viruses complemented with VSV G_Ind_ are denoted as G_Ind_* rVSV-ΔG-GFP, G*rVSV-ΔG-NiV_B_/F-GFP, and G* rVSV-ΔG-NiV_B_/G-GFP. To make the vaccine in Group 4 (Figure 1A), Vero cells were co-infected with G*rVSV-ΔG-NiV_B_/F-GFP and G* rVSV-ΔG-NiV_B_/G-GFP at MOI 5 for each virus. Supernatants were collected 24 h.p.i. and titered on BHK-21 cells complemented with VSV G_Ind_.

NiV_M_ #1999011924 was obtained from a patient from the 1999 outbreak in Malaysia (kindly provided by Dr. Thomas Ksiazek). NiV_M_ was chosen for challenge in ferrets based on our lethality data with this particular stock at the time of the study. The virus was propagated on Vero E6 cells in Eagle’s minimal essential medium (EMEM) supplemented with 10% fetal calf serum. The NiV_M_ challenge virus stock was assessed for the presence of endotoxin using The Endosafe®-Portable Test System (PTS) (Charles River, Wilmington, MA). Virus preparations were diluted 1:10 in Limulus Amebocyte Lysate (LAL) Reagent Water (LRW) per manufacturer’s directions and endotoxin levels were tested in LAL Endosafe®-PTS cartridges as directed by the manufacturer. Each preparation was found to be below detectable limits while positive controls showed that the tests were valid.

### Statistics

Conducting animal studies in BSL-4 severely restricts the number of animal subjects, the volume of biological samples that can be obtained and the ability to repeat assays independently and thus limit statistical analysis. Consequently, data are presented as the mean calculated from replicate samples, not replicate assays, and error bars represent the standard deviation across replicates.

### Animals

Animal studies were performed in BSL-4 biocontainment at the GNL at the UTMB at Galveston and were approved by the UTMB Institutional Animal Care and Use Committee (IACUC). Animal research was conducted in compliance with the Animal Welfare Act and other Federal statutes and regulations relating to animals and experiments involving animals and adheres to the principles stated in the eighth edition of the *Guide for the Care and Use of Laboratory Animals*, National Research Council, 2013. The facility where this research was conducted is fully accredited by the Association for Assessment and Accreditation of Laboratory Animal Care International.

Twenty female ferrets weighing 0.75-1 kg were housed in groups of 3 and 2 animals per vaccine group. Before vaccination, subjects were anesthetized by i.m. injection with ketamine-acepromazine-xylazine (KAX) cocktail and had transponder chips (BioMedic Data Systems, Seaford, DE) implanted subcutaneously for animal identification and temperature monitoring. For procedures, animals were anesthetized with KAX and vaccinated with ~ 1 × 10^7^ PFU by i.m. injection on day -28 (Figure 2A). Animals were inoculated intranasally (i.n.) with ~ 5 × 10^3^ pfu of NiV_M_ in 1 ml of Dulbecco’s minimal essential medium (DMEM) (Sigma-Aldrich, St Louis, MO) 28 days after vaccination (Figure 2A, *). Animals were anesthetized for clinical examination including temperature, respiration quality, blood collection, and on days 0, 6, and 21 p.c. Before and after challenge animals were assessed daily for weight, temperature, and scored on a scale of 0 of 9 for clinical observations based on coat appearance, social behavior, and provoked behavior; animals scoring 7 or greater were euthanized per IACUC protocol. Subjects in the vaccine cohorts were euthanized at the study endpoint on day 22 p.c. whereas the subjects in Group 1 had to be euthanized according to approved humane end points on day 7 or 8 p.c. All other subjects survived until the end of the study.

### Measurement of serum or plasma NiV F and G specific antibodies

Ferret serum collected at indicated time points was tested for IgG antibodies against NiV F and G using previously developed multiplexed microsphere assays [30]. 96-well filter plates were primed with PBS. Test sera were diluted in PBS at 1:10 for pre vaccination time points and 1:10,000 for time points after vaccination. Biotinylated goat anti-ferret IgG and streptavidin-phycoerythrin (strep-PE) were also diluted in PBS. Coupled microspheres (sG-HeV, sG-NiV, sF-Hev, sF-NiV) were prepared by sonication for 1 minute followed by vortex mixing for 1 minute each and then diluted in PBS. Priming liquid was removed from plates using a Bio-Plex Pro II Wash Station (Bio-Rad, Hercules, CA) and 100 μL containing 1500 of each coupled microsphere was added to each well. The microsphere mixture was removed by vacuum, 100 μL of diluted test sera was added to appropriate wells and incubated at room temperature (RT) for 30 minutes while shaking in the dark. Diluted test samples were removed by vacuum and 100 μL of diluted biotinylated goat anti-ferret (1:500) (Pierce, ThermoScientific, Rockford, IL) was added to each well and incubated as previously described above. Liquid was removed by vacuum and 100 μL of strep-PE (1:1000) (Qiagen, Valencia, CA) was added to each well and again incubated for 30 minutes. All liquid was removed from plates with a vacuum manifold and washed twice with 300 μL PBS, removing liquid between wash steps. Finally, 125 μL of PBS was added to each well and incubated for 2 minutes as described above. Samples were assayed for mean fluorescence intensity (MFI) across at least a 100 bead region performed on the BioPlex-200 machine and analyzed using Bio-Plex Manager Software (v 6.1) (Bio-Rad). MFI and the standard deviation (s.d.) of fluorescence intensity across 100 beads were determined for each sample and plotted.

### NiV_M_ serum neutralization assays

PRNTs were determined using a conventional serum neutralization assay. Briefly, sera were serially diluted twofold, and incubated with ~ 100 pfu of NiV for 1 hour at 37°C. Virus and antibodies were then added to individual wells of 6-well plates of confluent Vero cell monolayers. Plates were stained with neutral red 2 days after infection and plaques were counted 24 hours after staining. The 50% neutralization titer (PRNT_50_) was determined as the serum dilution at which there was a 50% reduction in plaque counts versus control wells.

### Specimen collection and processing in NiV-infected ferrets

Blood was collected and placed in MiniCollect EDTA tubes or serum tubes (Greiner Bio One, Monroe, NC). Immediately following sampling, 100 μl of blood was added to 600 μl of AVL viral lysis buffer (Qiagen) for RNA extraction. For tissues, approximately 100 mg was stored in 1 ml RNAlater (Qiagen) for 7 days to stabilize RNA. RNAlater was completely removed, and tissues were homogenized in 600 μl RLT buffer (Qiagen) in a 2-ml cryovial using a tissue lyser (Qiagen) and stainless steel beads. The tissues sampled included right lung upper lobe, right lung middle lobe, right lung lower lobe, left lung upper lobe, left lung middle lobe, left lung lower lobe, liver, spleen, kidney, adrenal gland, pancreas, and brain (frontal cortex). All blood samples were inactivated in AVL viral lysis buffer, and tissue samples were homogenized and inactivated in RLT buffer prior to removal from the BSL-4 laboratory. Subsequently, RNA was isolated from blood and swabs using the QIAamp viral RNA kit (Qiagen) and from tissues using the RNeasy minikit (Qiagen) according to the manufacturer’s instructions supplied with each kit.

### Hematology and serum biochemistry

Prior to the study, baseline blood and sera were collected via the anterior vena cava from all 20 ferrets. On days -28, 0, 6, and 21 blood was collected from all animals. Complete blood counts of total white blood cell counts, white blood cell differentials, red blood cell counts, platelet counts, hematocrit values, total hemoglobin concentrations, mean cell volumes, mean corpuscular volumes, and mean corpuscular hemoglobin concentrations were analyzed from blood collected in MiniCollect EDTA tubes (Greiner Bio One) using a Hemavet HV950FS instrument per manufacturer’s instructions (Drew Scientific, Oxford, CT). Serum analysis of blood chemistries was performed using a VetScan classic analyzer and comprehensive diagnostic profile rotors measuring of albumin (ALB), amylase, alanine aminotransferase (ALT), alkaline phosphatase (ALP), calcium, glucose, total protein, total bilirubin (TBIL), blood urea nitrogen (BUN), creatinine (CRE), phosphorus, sodium, and total protein (Abaxis, Union City, CA). All blood and serum samples were processed and analyzed immediately after collection.

### Histopathology and immunohistochemistry

Necropsy was performed on all subjects. Tissue samples of all major organs were collected for histopathologic and immunohistochemical examination and were immersion-fixed in 10% neutral buffered formalin for at least 21 days in BSL-4. Subsequently, formalin was changed; specimens were removed from BSL-4, processed in BSL-2 by conventional methods and embedded in paraffin and sectioned at 5 μm thickness. For immunohistochemistry, specific anti-NiV immunoreactivity was detected using an anti-NiV N protein rabbit primary antibody (kindly provided by Dr. Christopher Broder) at a 1:5000 dilution for 30 minutes. The tissue sections were processed for immunohistochemistry using the Dako Autostainer (Dako, Carpinteria, CA). Secondary antibody used was biotinylated goat anti-rabbit IgG (Vector Laboratories, Burlingame, CA) at 1:200 for 30 minutes followed by Dako LSAB2 streptavidin-HRP (Dako) for 15 minutes. Slides were developed with Dako DAB chromagen (Dako) for 5 minutes and counterstained with hematoxylin for one minute. Non-immune rabbit IgG was used as a negative staining control.

### Detection of NiV load

RNA was isolated from blood or tissues and analyzed using primers/probe targeting the N gene and intergenic region between N and P of NiV for quantitative real-time PCR (qRT-PCR) with the probe used here being 6-carboxyfluorescein (6FAM)-5′ CGT CAC ACA TCA GCT CTG ACG A 3′-6 carboxytetramethylrhodamine (TAMRA) (Life Technologies, Carlsbad, CA). NiV RNA was detected using the CFX96 detection system (Bio-Rad) in One-step probe qRT-PCR kits (Qiagen) with the following cycle conditions: 50°C for 10 minutes, 95°C for 10 seconds, and 40 cycles of 95°C for 10 seconds and 59°C for 30 seconds. Threshold cycle (*CT*) values representing NiV genomes were analyzed with CFX Manager Software, and data are shown as genome equivalents (GEq). To create the GEq standard, RNA from NiV challenge stocks was extracted and the number of NiV genomes was calculated using Avogadro’s number and the molecular weight of the NiV genome. Virus titration was performed by plaque assay with Vero cells from all serum and control tissue samples. Briefly, increasing 10-fold dilutions of the samples were adsorbed to Vero cell monolayers in duplicate wells (200 μl); the limit of detection was 25 pfu/ml.

## Competing interests

The authors declare that they have no competing interests.

## Authors’ contributions

CEM designed the vaccines and vaccination study, carried out the animal vaccination and infection studies, processed animal tissues and blood, analyzed data, and drafted the manuscript. KMV cloned, recovered, characterized, and propagated the vaccines, processed animal tissues and blood, and participated in manuscript preparation. RWC carried out animal vaccination and infection studies, processed animal tissues and blood, analyzed data, and participated in manuscript preparation. KNA participated in animal infection studies, processed animal tissues and blood, performed serology, performed virus isolation, performed qRT-PCR, analyzed data, and participated in manuscript preparation. KAF participated in animal infection studies, provided veterinary pathology expertise, analyzed data, and participated in manuscript preparation. MAW participated in design of vaccine construction and study design, analyzed data, and edited manuscript. TWG conceived the study, supported the work with research funds from the National Institutes of Health (U01 AI082121) and UTMB, analyzed data, and edited the manuscript. All authors read and approved the final manuscript.
